# A microRNA Link to Glioblastoma Heterogeneity

**DOI:** 10.3390/cancers4030846

**Published:** 2012-09-03

**Authors:** Sanjay K. Singh, Alenoush Vartanian, Kelly Burrell, Gelareh Zadeh

**Affiliations:** The Arthur and Sonia Labatt Brain Tumor Research Centre, Hospital for Sick Children, University of Toronto, Toronto, Ontario M5G 1L7, Canada; E-Mails: sanjay.singh@sickkids.ca (S.K.S.); a.albertvartanian@mail.utoronto.ca (A.V.); kelly.burrell@sickkids.ca (K.B.)

**Keywords:** glioma, glioblastoma, microRNA, angiogenesis, glioma stem cells, metabolism, Warburg effect

## Abstract

Glioblastomas (GBM) are one of the most malignant adult primary brain tumors. Through decades of research using various model systems and GBM patients, we have gained considerable insights into the mechanisms regulating GBM pathogenesis, but have mostly failed to significantly improve clinical outcome. For the most part GBM heterogeneity is responsible for this lack of progress. Here, we have discussed sources of cellular and microenvironmental heterogeneity in GBMs and their potential regulation through microRNA mediated mechanisms. We have focused on the role of individual microRNAs (miRNA) through their specific targets and miRNA mediated RNA-RNA interaction networks with the potential to influence various aspects of GBM heterogeneity including tumor neo-vascularization. We believe a better understanding of such mechanisms for regulation of GBM pathogenesis will be instrumental for future therapeutic options.

## 1. Introduction

Tumors are increasingly being viewed as complex tissue, which comprises of a heterogeneous mix of distinct cell types including cancer cells and recruited normal cells [1]. The current emphasis of tumor biology research is focused on understanding the interplay of cancer cells and tumor associated stroma and how these interactions contribute to tumorigenesis. In this review we have focused on the most common and malignant adult primary brain tumor, glioblastoma (GBM). In spite of our greater understanding of the biology of GBMs, GBM patients have seen only moderate improvements in outcome, as their median survival with best available treatment remains at 12–16 months [2]. It is widely accepted that the future treatment options for GBMs will greatly benefit from our improved understanding of the complex interactions between cancer cells and components of tumor microenvironment [3,4,5,6,7,8,9,10,11,12,13]. GBMs are characterized by significant intra-tumor regional heterogeneity comprised of areas of necrosis surrounded by a pseudopalisading proliferative edge along with a highly vascular stroma. These histological diverse regions differ in factors including the composition of subpopulation of cells, such that cells from the inner core of the tumor with higher hypoxic gradient have been shown to harbor higher concentration of glioma stem cells (GSCs) and show enhanced temozolomide (TMZ) resistance [14]. Furthermore, central and peripheral regions differ in their apoptotic index, hypoxia, migratory potential, vascularity and the therapeutic resistance, increasing the level of complexity to the microenvironmental/metabolic heterogeneity in GBMs. Here we have focused on cellular heterogeneity, their interactions in tumor microenvironment and their physiological or metabolic status as components of GBM heterogeneity. Specifically we have discussed function of individual microRNAs (miRNA) through their specific targets and miRNA mediated RNA-RNA interaction networks with potential to influence various aspects of GBM heterogeneity including tumor neo-vascularization.

We have discussed the role of miRNAs in multiple aspects of GBM biology and their clinical and functional significance in regulating factors such as angiogenesis, metabolic reprogramming and stem cell behavior. MiRNAs can function by modulating vascular integrity and angiogenesis, which is a known hallmark of high-grade gliomas, also they can alter the metabolic phenotype of GBMs by regulating key metabolic enzymes, tumor hypoxia, lactate production or signaling pathways that regulate aerobic glycolysis. In addition, miRNA have been shown to have a functional relevance in regulation of critical genes and parameters implicated in GSC behavior and differentiation.

Tumor heterogeneity arises in a multitude of ways, either as a consequence of an individual or combination of factors. For instance factors contributing to tumor heterogeneity includes: clonal evolution of cancer cells that have undergone genetic and/or epigenetic changes [15,16], functional and phenotypic changes in cancer cells under the influence of different microenvironmental conditions such as hypoxia [17], and finally, cancer stem cells which while maintaining their own population can give rise to non-tumorogenic differentiated cancer cells [18,19,20,21]. GBMs have been shown to harbor glioma stem cells (GSCs) with tumorigenic potential, non-tumorigenic cancer cells, and other recruited cells; together they form heterogeneous microenvironmental niches where chemo- and radio-resistant GSCs have been shown to reside [4,13,22,23,24].

## 2. Glioma Stem Cells (GSCs)

Although there are no universal marker(s) for identification/enrichment of GSCs, there is growing consensus that there is a sub-population of cells in GBMs, which are selectively tumorigenic in orthotopic tumor models and share properties of normal stem/progenitor cells. Functional features of GSCs include the ability to propagate histopathologically similar tumors in orthotopic models, ability to self-renew through symmetric and asymmetric cell division and retain indefinite proliferation capacity. Apart from these essential functional criteria, GSCs have also been commonly shown to be both chemo- and radiation resistant [4,5,11], selectively populated in specific niches (e.g., perivascular niche (PVN)) within the tumor [6,25,26], immunosuppressive [27] and angiogenic [12]. In accordance with hierarchical cancer stem cell model, the GSCs population within a tumor may have varying degree of stemness ranging from quiescent (high label retaining [28], equivalent to adult neural stem cells), proliferating progenitors (transient amplifying population) as well as differentiated cancer cells. Thus, various aspects of GBM pathogenesis can be orchestrated by GSCs function, as they may be responsible for the establishment of cellular heterogeneity, through paracrine and autocrine signaling within the PVN to increase neo-vascularization, facilitate recruitment of cells of various origins, and maintain self-renewal. It is also notable that the PVN harbors chemo- and radio-resistant GSCs or tumor cells. GSCs have also been shown to produce pro-angiogenic factors such as vascular endothelial growth factor (VEGF) and express its cognate receptor VEGFR, thus forming an autocrine signaling loop. Blocking VEGFR in GSCs has been shown to decrease GSC proliferation and sensitize them to cell death in response to radiotherapy [29]. GSCs share some properties with neural precursor cells (NPCs). However, functionally when NPCs are present in tumor microenvironment, they harbor anti-tumor properties [30,31,32,33,34]. In GBM patients where young age has been associated with better prognosis, the role of NPCs is of particular interest, as this correlates with the abundance of NPCs in the brain [32], which decrease with age [35]. It has been demonstrated that NPC are able to migrate towards and infiltrate the primary glioma tumor site and also track along the invasive tumor cells and induce tumor cell death by releasing endogenous anti-tumor signals such as Cyclin D1 and D2 [34,36]. It has been suggested that signaling molecules generated from the tumors such as stromal cell-derived factor-1 (SDF-1) or VEGF provides the attractive force for NPS to migration away from the subventricular zone (SVZ) to the tumor mass. This process is age dependent and with increased age NPCs have reduced ability to migrate and produce anti-tumor signals. Results from various groups have demonstrated that GSCs are flexible in terms of their cross-lineage differentiation potential with a portion of endothelial cells (EC) within the tumor reportedly being derived from GSCs [37,38,39,40]. Thus, GSCs’ contribution to GBM heterogeneity is of growing interest for understanding GBM pathobiology with clinical implications.

## 3. Tumor Associated Vasculature

Tumor vasculature plays a critical role in the maintenance, growth and progression of tumors, as they are largely responsible for oxygen and nutrient supply to the tumor. GBMs are one of the most highly vascularized solid human cancers [41]. The tumor-associated vasculature in GBMs is pathological, with hyper-proliferative and piling of ECs forming glomerular tufts [42,43]. ECs together with pericytes and vascular smooth muscle cells are intricately involved in this hyperproliferation of microvasculature [44]. These hyperproliferative areas within tumor may overlap with PVN, which in turn are important for maintenance of chemo- and radio-resistant brain tumor cells including GSCs [6,25,26]. ECs have been shown to be involved in extensive cross-talk with cells in the PVN [6]. The most potent pro-angiogenic factor, VEGF, is known to be produced by GSCs amongst other tumor cells, which in turn can regulate extent of tumor neo-vascularization through stimulation of ECs proliferation. Significance of this important paracrine stimulus is evident as anti-VEGF treatment results in decreased tumor neo-vascularization and growth of gliomas in xenograft models in preclinical studies and similar encouraging results have been demonstrated in recent clinical trials [5,7,45]. It has been shown that ECs secrete factors responsible for maintenance of “stemness” of GSCs in PVN [6]. Specifically, in platelet-derived growth factor (PDGF) sub-group of gliomas, nitric oxide (NO) producing enzyme eNOS is upregulated in vascular endothelium and this secreted NO diffuses into the PVN in turn helping to maintain Notch dependent self-renewal and tumorigenic potential of GSCs [25]. Additionally, in sonic hedgehog (shh) dependent GBMs, shh produced by tumor associated ECs have been shown to play a role in maintenance of GSCs [46,47,48,49]. ECs are also responsible for recruitment of pericytes to neovascularization sites resulting in the establishment of new basement membrane [50]. Recently, hypoxia-inducible factor (HIF1α) has been shown ro mediate the recruitment of pericytes to the PVN resulting in neovascularization in GBM has been documented [51]. Together with vascular smooth muscle cells, pericytes provide structural support to vasculature and lack of pericytes from vasculature results in leaky blood vessels [52,53].

## 4. GBM Heterogeneity Orchestrated by Non-Physiological Levels of Oxygen, pH, and Metabolites

Recently, the implications and better understanding of cancer energy metabolism has regained traction since the first observations by Otto Warburg that glucose metabolism in cancer cells is fundamentally different from that which occurs in normal tissue [54]. Warburg observed excessive “fermentation” of glucose to lactate in cancer cells, even when enough oxygen was present for normal respiration to take place. Although he mistakenly contributed the altered metabolism to irreversibly damaged mitochondria and hence “the origin of cancer cell”, nevertheless his contributions were pivotal to our early understanding of cancer metabolism. Now we can appreciate that metabolic reprograming is possibly an adaptive response to meet the challenges of rapidly proliferating tumor cells [55]. The explanation for the switch to aerobic glycolysis or the Warburg effect as it is commonly referred to, is complex and as shown by Vander Heiden *et al*. [55] aerobic glycolysis satisfies the metabolic requirement of cancer cells by providing biosynthetic precursors, and that the high flux of substrates down the glucose pathway provides carbon source needed for macromolecular production such as lipids, nucleotides and proteins [55]. The metabolic adaptation in tumor microenvironment can be influenced by hypoxia, pH and/or the status of oncogenes and tumor suppressors; the specific role of these factors and their interactions in the establishment of the metabolic phenotype in tumor is still debated [56]. Altered expression of glucose transporters (GLUTs) and metabolic enzymes such as pyruvate kinase (PK), hexokinase II (HK2), lactate dehydrogenase (LDH), pyruvate dehydrogenase kinase (PDK) have been demonstrated in various cancers including GBMs. The expression of embryonic PK (M2) isoform is prevalent in primary tumor samples and cell lines [57,58]. PKM2 provides a metabolic and growth advantage by slowing glycolysis to allow shunting of carbon metabolites to key subsidiary biosynthetic pathways [58]. Likewise, HK2 is upregulated in cancer cells and plays a key role in regulating the Warburg effect in GBMs. Wolf *et al*. [59] showed that stable loss of HK2 inhibited aerobic glycolysis and led to an increase in normal oxidative respiration, decrease in lactate production and overall a switch to oxidative phosphorylation. Conversion of pyruvate (by product of glycolysis or glutaminolysis) to lactate is a reversible step governed by lactate dehydrogenase enzyme [60]. Enhanced LDHA expression is reported in tumors to facilitate maintenance of glycolysis by reducing intracellular lactate levels, and a concomitant increase in extracellular lactate and acidification which can promote invasion and dampening of immune surveillance [60,61]. It is also proposed that lactate can re-enter cells within tumor and act as a fuel for oxidative phosphorylation in oxygenated areas (reverse Warburg). PDK is another perpetrator of aerobic glycolysis in gliomas. It can inhibit pyruvate entry to TCA cycle by inactivation of pyruvate dehydrogenase (PDH) enzyme. Dichloroacetate (DCA) showed favorable results in phase II clinical trial in GBMs by inhibiting PDK and facilitating the switch to normal mitochondrial respiration [62].

Other molecular mechanisms involving non-metabolic genes such as known oncogenes and tumor suppressors have been implicated to converge and regulate different steps of metabolic pathways. These regulations orchestrate a state of high glucose uptake/flux, activate genes involved in the glycolysis (PI3K/AKT/P53 pathway), upregulate glutaminolysis (P53, MYC) or lipid synthesis (PI3K/AKT) and alleviate glycolysis repression (P53) [55]. The interplay of metabolic enzymes with transcriptional regulators and tumor metabolites may establish a heterogeneous metabolic microenvironment within different grades of gliomas. Since GBMs are known to reside in a heterogeneous metabolic microenvironment, this can impact the development, progression and survival of GBM patients. Inadequate vascular function, metabolic reprogramming, hypoxia, oxidative stress, acidity or pH imbalance in combination with GBMs intra-tumor regional heterogeneity (including genetic heterogeneity [63]) are some of many factor which has to be take into consideration during the prognostication, treatment, therapy stratification and therapeutic resistance.

## 5. Tumor Hypoxia

Tumor hypoxia has been recognized as one of the most prominent features of the dynamic neoplastic microenvironment. Within tumor hypoxia is dynamic [64]. In addition to the diffusion-limited hypoxia [64], the chaotic and disorganized tumor vascular network leads to fluctuating changes in pO2. Thus, solid tumors frequently harbor population of cancer cells with dynamic oxygen gradients that can drive and maintain aggressive tumor behavior, genetic instability, increased invasion, angiogenesis, and resistance to conventional therapy [64]. Indeed a hallmark feature of GBM histopathology is extensive cellular necrosis, presented as foci of micronecrosis encircled by pseudopalisading hypercellular neoplasmic cells [42,65,66,67]. Hypoxia is mostly present in high-grade gliomas, with GBMs dominated with moderate to severe hypoxic cells with a subset of oxic cells [4,5,30]. Evans *et al*. have demonstrated GBMs’ dynamic oxygenation with oxygen concentrations ranging between 2.5% and 0.5% for mild hypoxia and 0.5%–0.1% for moderate/severe hypoxia [68]. Hypoxic heterogeneity within a tumor may explain the observed variations in cell death, radio-resistance and therapeutic response [64]. It has been postulated that differential growth of tumor and ECs result in an imbalance in supply and demand where oxygen and nutrient requirement of the tumors are not met. Low oxygen tension or hypoxia (chronic and acute hypoxia or anoxia) has been shown to have different effect on cellular processes. One of the major players in hypoxia adaptation is the activation and stabilization of HIF1α, 2α and 3α and induction of slew of downstream genes with a broad functional consequence in various cancers [69]. HIFs has been recognized to play a central role in GBMs adaptation to harsh microenvironmental condition by regulating genes involved in aerobic glycolysis, angiogenesis, cell survival/death, pH regulation, invasion and metastasis and stem cell maintenance [70,71]. The most fundamental result of HIF activation is a shift in energy consumption and production by increasing aerobic glycolysis and angiogenesis, ultimately resulting in regulation of oxygen delivery and consumption [71,72]. Additional factors have been recognized that can lead to HIF1α activation and accumulation in normoxic conditions (pseudohypoxia): include oncogenic mutations, mitochondrial ROS production and accumulation of TCA cycle enzymes [72]. The extent of hypoxia heterogeneity and resulting cellular and molecular alterations in GBMs thus has prognostic implications.

## 6. Identifying Underlying Mechanisms for Diverse Events in Heterogeneous Tumors like GBMs

In the era of systems biology, cancer is viewed as a manifestation of an interconnected network of intra- and extra-cellular events, which co-exist to establish and maintain the tumor microenvironment. At a molecular level, a network of transcription factors have been described, which regulate both normal (in normal development) and abnormal (in cancers and various diseases) cellular behavior [73,74,75,76]. Recently an extensive molecular network involving miRNAs have been described in GBM using most exhaustive expression database The Cancer Genome Atlas (TCGA) [77]. Together with experimentally validated role of miRNAs [78] and mRNA-miRNA-network [77], miRNA mediated mechanisms have potential to be that elusive layer of regulation, which may help explain the heterogeneous events important for establishment and maintenance of tumor microenvironment.

## 7. microRNA: Biogenesis and Functions

MicroRNAs are small (17–25 bases) non-coding RNAs which influence the expression of the majority of genes in the eukaryotic genome. This miRNA-mediated regulation of genes thus constitutes another layer of post-transcriptional gene regulation. The majority of miRNAs are transcribed in an RNA polII dependent manner [79,80], while some, such as the cluster of miRNAs on chromosome 19, have been shown to be transcribed by RNA polIII [79]. Many miRNA genes are transcribed as polycistronic transcripts, as they tend to be clustered together in a specific chromosomal location [80]. Some miRNA genes (e.g., mmu-mir-127 and mmu-mir-136) have also been reported to reside in imprinted loci in the genome and thus expressed from maternally inherited chromosome [81]. The primary miRNA transcripts (pri-miRNAs) have conserved secondary structures (stem-loop with 5' and 3' tails), which are recognized and processed by endoribonucleases complexes in the nucleus and cytoplasm. The nuclear endoribonuclease Drosha and DGCR8 (microprocessor complex) excise the stem-loop of approximately 70 nucleotides with a 2-nucleotide (nt) 3' overhang to produce pre-miRNA [82]. However, mirtron are processed by splicing machinery and do not require microprocessor activity for their maturation steps in nucleus but follow similar maturation steps in cytoplasm as the rest of the miRNAs [83,84]. These pre-miRNAs and mirtrons are then exported out of nucleus by RanGTP and Exportin-5 complex in a GTP-dependent manner [85,86,87]. In the cytoplasm, the pre-miRNA stem loop is further processed by the Dicer. The Dicer forms a complex with pre-miRNA, the two RNaseIII domains of Dicer cleave the pre-miRNA separated by 2 nucleotides roughly about 22 nucleotides away from the termini generating two nucleotide overhang at the 3' ends [88]. Functional miRNA-ribonucleoprotein complex is known as the miRNA-RNA-Induced Silencing Complex (miRNA-RISC). The miRNA duplex produced after Dicer activity then unwinds and binds to Argonaute (Ago) protein to form the core of the functional miRNA-RISC [89]. Argonaute family member AGO2 has RNaseH-like PIWI (P-element-induced wimpy testis) domain and is capable of cleavage of target RNA at the center of siRNA-mRNA pair [90,91,92]. The interaction between miRNP complex and its target mRNA is RNA guided and involves specific base-pairing interactions between miRNA and partially complementary sequences located (miRNA response elements, MREs) in the target RNA (e.g., 3'UTR). Specifically, the extent of complementarity to miRNA positions 2 and 8 (seed sequences/region) defines the affinity of miRNA-target RNA interaction [93,94,95,96,97]. The mechanisms of miRNA mediated repression includes translational repression, mRNA de-adenylation and degradation, and sequestration of mRNA to sub-cellular bodies [98].

## 8. miRNA Targets with Established Role in Gliomagenesis

miRNAs are mostly negative regulators of gene expression. Based on their expression levels and the genes they target in a given cellular context, miRNAs can broadly be grouped as oncogenic miRNAs or tumor suppressor miRNAs. Work from various groups studying individual miRNA function or global expression profiles of miRNA in gliomas, has established that miRNAs do in fact play a crucial role in different aspects of gliomagenesis. In gliomas, miRNA mediated mechanisms regulate a range of cellular functions which include: cell viability, cell proliferation, cell migration and invasion, apoptosis, tumor growth, cell cycle, chemo- and radio resistance, angiogenesis, tumor metabolism, and maintenance of “stemness” status of GSC [99]. Table 1 shows miRNAs involved in various aspects of GBMs and their relevant targets. The oncogenic miRNAs with known function/targets in gliomas (studied in tumors and/or glioma cells) include: miR-21, miR-221/222, miR-296, miR-10b, miR-17, miR-195, miR-455-3p, miR-10a*, miR-182, miR-451, miR-17-92 cluster *etc*.; and miRNAs with tumor suppressor activity include: miR-181, miR15b, miR-146b, miR-125b, miR-153, let-7, miR-153, miR-184, miR-7, miR-137, miR-128, miR-34a *etc*. [99]. Multiple signaling pathways that are deregulated in gliomas such as P53-, TGFβ-, Apoptotic-, Interferon (IFNα/IFNβ)-, Notch-, NF-κB-, EGFR-, and PTEN/PI3K/AKT-pathways could potentially be targeted by miRNAs [100]. Some miRNAs have more implications in GBM pathogenesis due to their wider targets, more robust miRNA binding sites, broader functional coverage and their multiple roles when compared to other miRNAs. More consideration should be given to these miRNAs, miRNA-221/222 in having a potential role in angiogenesis, invasiveness and resistance to therapy [101,102]) due to their broader functional relevance. Table 2 is compiled using experimentally validated miRNA-mRNA pairs and target genes were grouped according to their known function in specific pathways using DAVID [103,104]. The miRNAs with known levels in GBMs are color coded [105]. In the following sections we have attempted to highlight pro- and anti-tumorigenic function of miRNAs and how their aberrations can mechanistically modulate multiple aspects of GBMs.

**Table 1:** List of miRNAs discussed in the text with their relevant functions and targets in GBMs or other cancers.

MicroRNA functions	MicroRNAs	Relevant targets	Reference
Glioma stem cells	miR-124	PTBP1, CDK6, SCP1, LAMC1, ITGB1	[106,107,108,109,110,111,112,113]
	miR-137	CDK6	
	miR-128	Bmi-1, E2F3A	
	miR-7	EGFR, IRS2	
	miR-425		
	miR-486		
	miR-451		
	miR-34a	Notch1, Notch2	
	miR-326	Notch	
Pro-agiomiRs	miR-126,	Spred-1, PIK3R2	[114,115,116,117,118,119,120,121,122,123,124,125,126]
	miR-17-92 cluster	TSP-1	
	miR-378	Sufu, Fus-1	
	miR-296	HGS	
	miR-21		
	miR-210	Ephrin-A3	
	miR-130a	HOXA5, GAX	
	miR-125b	MAZ	
	miR-101	EZH2	
Anti-angiomiRs	miR 221/222	c-Kit	[127,128]
	miR-320	IGF-1	
Aerobic glycolysis & related signaling pathways	miR-326	PKM2	[129,130,131]
	miR-21	PI3K/AKT/P53	
	miR-26a,	PI3K/AKT	
	miR-221/222	PI3K/AKT	
	miR-451	LKB1/AMPK	
	miR-128	AKT	
	miR-25	P53	
	mir-32	P53	
	miR-34a	MYC	
	miR-155/143	CEBPb	
	mir-23a/b	MYC	
Hypoxamirs	miR-20b	Sirt1	[132,133,134,135]
	miR-210	ISCU1/2	
	miR-199a	HIF-1α, Sirt1	

**Table 2:** Experimentally validated targets of miRNAs are components of pathways known to play key role in GBM biology. MicroRNAs shown in underline are over expressed and shown in bold are under expressed miRNAs in GBMs.

Glioma de novo pathway (KEGG)			
**S. No.**	**Gene name**	**miRNA(s)**	
1	*PDGFA*	hsa-let-7d	
2	*PDGFB*	hsa-miR-146b-3p	
3	*EGFR*	**hsa-miR-1, hsa-miR-128**, hsa-miR-146a, hsa-miR-16, hsa-miR-21,** hsamiR-7**	
4	*PDGFRA*	hsa-let-7b	
5	*PDGFRB*	hsa-miR-224	
6	*IGF1R*	**hsa-miR-7**, hsa-miR-122, **hsa-miR-133b, hsa-miR-138**, hsa-miR-145, hsamiR-194	
7	*GRB2*	**hsa-miR-433**	
8	*CALM2, CALM3*	**hsa-miR-1**	
9	*CAMK2G*	**hsa-miR-219-5p**	
10	*HRAS*	hsa-let-7a, hsa-miR-143, hsa-miR-181a,	
11	*KRAS*	hsa-let-7a, hsa-let-7g, hsa-miR-143, hsa-miR-155, hsa-miR-181c, hsa-miR-18a*, hsa-miR-96	
12	*NRAS*	hsa-let-7a, hsa-let-7b, hsa-let-7c, hsa-miR-20a	
13	*PIK3R1*	**hsa-miR-29a**	
14	*PIK3R2*	hsa-miR-126	
15	*PTEN*	hsa-miR-106b, hsa-miR-141, hsa-miR-17, hsa-miR-18a, hsa-miR-19a, hsa-miR-19b, hsa-miR-20a, hsa-miR-21, hsa-miR-214, hsa-miR-216a, hsa-miR-217, **hsa-miR-221**, hsa-miR-222, hsa-miR-26a, hsa-miR-494	
16	*ARAF*	**hsa-miR-124**	
17	*RAF1*	hsa-miR-125b, **hsa-miR-7**	
18	*AKT1*	hsa-miR-125b, hsa-miR-149*, hsa-miR-185, hsa-miR-451	
19	*MAP2K1*	**hsa-miR-34a**, hsa-miR-424	
20	*MAPK1*	hsa-miR-199b-3p	
21	*CDKN2A*	hsa-miR-24, hsa-let-7g, hsa-miR-125b	
22	*TP53*	hsa-miR-25, hsa-miR-30d, hsa-miR-612, **hsa-miR-125a-5p**, hsa-miR-125b, hsa-miR-1285, hsa-miR-15a, hsa-miR-16, **hsa-miR-221**, hsa-miR-222	
23	*CDKN1A*	hsa-miR-106a, hsa-miR-106b, **hsa-miR-125a-5p, hsa-miR-132**, hsa-miR-145, hsa-miR-146a, hsa-miR-146b-5p, hsa-miR-17, hsa-miR-182, hsa-miR-208a, hsa-miR-208b, hsa-miR-20a, hsa-miR-20b, hsa-miR-28-5p, hsa-miR-298, **hsa-miR-299-5p**, hsa-miR-302a, hsa-miR-345, hsa-miR-363, hsa-miR-372, hsa-miR-423-3p, hsa-miR-503, hsa-miR-515-3p, hsa-miR-519d, hsa-miR-519e, hsa-miR-520a-3p, hsa-miR-520b, hsa-miR-520h, hsa-miR-572, hsa-miR-639, hsa-miR-654-3p, hsa-miR-657,hsa-miR-93, hsa-miR-942, hsa-miR-96	
24	*CDKN2A*	hsa-let-7g, hsa-miR-125b, hsa-miR-24	
25	*CCND1*	hsa-let-7b, hsa-miR-106b, hsa-miR-15a, hsa-miR-15b, hsa-miR-16, hsa-miR-16-1*, hsa-miR-17, hsa-miR-193b, hsa-miR-19a, hsa-miR-20a, hsa-miR-302a, hsa-miR-302c, **hsa-miR-34a**, hsa-miR-424, hsa-miR-449a, hsa-miR-503	
26	*CDK4*	hsa-miR-124, hsa-miR-145, hsa-miR-24, hsa-miR-302a, **hsa-miR-34a**, hsa-miR-34b, hsa-miR-34b*, hsa-miR-34c-5p	
27	*CDK6*	hsa-miR-124, **hsa-miR-137**, hsa-miR-16, hsa-miR-185, **hsa-miR-203, hsa-miR-29a, hsa-miR-29b, hsa-miR-29c**, hsa-miR-30a*, **hsa-miR-34a**, hsa-miR-34b, hsa-miR-34b*, hsa-miR-424, hsa-miR-449a	
28	*RB1*	hsa-miR-106a, hsa-miR-106b, hsa-miR-17, hsa-miR-20a, hsa-miR-23b, hsa-miR-26a, hsa-miR-335, hsa-miR-675	
29	*E2F1*	hsa-let-7a, hsa-miR-106a, hsa-miR-106b, hsa-miR-126, hsa-miR-149*, hsa-miR-17, hsa-miR-20a, hsa-miR-21, hsa-miR-223, hsa-miR-23b, **hsa-miR-330-3p, hsa-miR-34a**, hsa-miR-93, hsa-miR-98	
**TGF beta Signaling pathway (KEGG)**			
**S. No.**	**Gene name**	**miRNA(s)**	
1	*BMP7*	hsa-miR-22, hsa-miR-342-3p	
2	*THBS1*	hsa-let-7a, hsa-let-7b, **hsa-miR-1**, hsa-miR-17, hsa-miR-20a, hsa-miR-30a*, hsa-miR-92a, hsa-miR-98	
3	*TGFB1*	hsa-miR-24	
4	*TGFB2*	hsa-miR-141	
5	*TGFB3*	hsa-miR-29a	
6	*LEFTY1, LEFTY2*	hsa-miR-302a, hsa-miR-302d	
7	*BMPR1B*	hsa-miR-125b	
8	*BMPR2*	**hsa-miR-129-5p**, hsa-miR-17, hsa-miR-19a, hsa-miR-19b, hsa-miR-20a, hsa-miR21, hsa-miR-92a	
9	*TGFBR1*	hsa-let-7c, **hsa-miR-128**, hsa-miR-204	
10	*TGFBR2*	hsa-miR-17, hsa-miR-18a, hsa-miR-19a, hsa-miR-19b, hsa-miR-204, hsa-miR-20a, hsa-miR-21, hsa-miR-302b, hsa-miR-372, hsa-miR-590-5p, hsa-miR-92a	
11	*ACVR1*	hsa-miR-197	
12	*ACVR2A*	hsa-miR-16	
13	*ACVR1C*	hsa-miR-122, hsa-miR-147, hsa-miR-22, hsa-miR-376a, hsa-miR376a*, hsa-miR-376b, hsa-miR-376c, hsa-miR-412	
14	*SMAD1*	hsa-miR-155, hsa-miR-26a	
15	*SMAD2*	hsa-miR-155	
16	*SMAD5*	hsa-miR-155	
17	*RHOA*	hsa-miR-122, hsa-miR-155, hsa-miR-185, hsa-miR-31	
18	*ROCK1*	hsa-miR-146a, hsa-miR-584	
19	*ROCK2*	**hsa-miR-138**	
20	*SMAD4*	hsa-miR-17, hsa-miR-18a, hsa-miR-19a, hsa-miR-20a, hsa-miR-26a, hsa-miR-483-3p, hsa-miR-92a	
21	*MAPK1*	hsa-miR-199b-3p	
22	*ID1*	hsa-miR-100, hsa-miR-520h	
23	*ID3*	hsa-miR-520h	
24	*RBL1, RBL2*	hsa-miR-106b, hsa-miR-17, hsa-miR-20a	
25	*E2F5*	hsa-let-7b, hsa-miR-192, **hsa-miR-34a**	
26	*CREBBP*	hsa-miR-324-3p	
27	*EP300*	hsa-miR-182, hsa-miR-194, hsa-miR-200b, hsa-miR-200c, hsa-miR-26b, hsa-miR-374a, hsa-miR-429	
28	*SP1*	**hsa-miR-124, hsa-miR-218, hsa-miR-29b**	
29	*MYC*	hsa-let-7a, hsa-let-7g, hsa-miR-145, hsa-miR-17, hsa-miR-20a, hsa-miR-21, hsa-miR-24, hsa-miR-26a, **hsa-miR-34a**, hsa-miR-34b, hsa-miR-34b*, hsa-miR-34c-5p, hsa-miR-378, hsa-miR-98	
**Notch signaling pathway (KEGG)**			
**S. No.**	**Gene name**	**miRNA(s)**	
1	*DLL1*	**hsa-miR-34a**	
2	*JAG1*	hsa-miR-200c, hsa-miR-21, **hsa-miR-34a**	
3	*ADAM17*	hsa-miR-122	
4	*NOTCH1*	**hsa-miR-129-5p**, hsa-miR-144, hsa-miR-30a, **hsa-miR-326, hsa-miR-34a**	
5	*NOTCH2*	**hsa-miR-1**, hsa-miR-16, hsa-miR-181c, **hsa-miR-326, hsa-miR-34a**	
6	*NOTCH3*	hsa-miR-206	
7	*NOTCH4*	hsa-miR-181c	
8	*DVL2*	hsa-miR-324-3p	
9	*NUMB*	hsa-miR-31	
10	*NUMBL*	hsa-miR-122	
11	*PSEN1*	hsa-miR-562	
12	*CREBBP*	hsa-miR-324-3p	
13	*EP300*	hsa-miR-182, hsa-miR-194, hsa-miR-200b, hsa-miR-200c, hsa-miR-26b, hsa-miR374a, hsa-miR-429	
14	*KAT2B*	hsa-miR-106b, hsa-miR-181a, hsa-miR-181b, hsa-miR-19a, hsa-miR-19b, hsa-miR-25, hsa-miR-32, hsa-miR-92a, hsa-miR-93	
15	*CTBP1*	**hsa-miR-137**	
16	*HDAC1*	hsa-miR-449a	
17	*NCOR2*	hsa-miR-10a, hsa-miR-10b	
18	*HES1*	hsa-miR-199b-5p, hsa-miR-23a	
**VEGF signaling pathway (KEGG)**			
**S. No.**	**Gene name**	**miRNA(s)**	
1	*VEGFA*	hsa-miR-106a, hsa-miR-106b, **hsa-miR-107**, hsa-miR-125a-5p, hsa-miR-126, hsa-miR-134, hsa-miR-140-5p, hsa-miR-147, hsa-miR-150, hsa-miR-15a, hsa-miR-15b, hsa-miR-16, hsa-miR-17, hsa-miR-195, hsa-miR-205, hsa-miR-20a, hsa-miR-20b, hsa-miR-29b, hsa-miR-302d, hsa-miR-330-3p, **hsa-miR-34a**, hsa-miR-34b, hsa-miR-361-5p, hsa-miR-372, hsa-miR-373, hsa-miR-378, **hsa-miR-383, hsa-miR-504**, hsa-miR-520g, hsa-miR-520h, hsa-miR-93	
2	*CDC42*	**hsa-miR-137**, hsa-miR-185, hsa-miR-216a, hsa-miR-224, hsa-miR-330-3p, hsa-miR-608	
3	*PIK3R1*	**hsa-miR-29a**	
4	*PIK3R2*	hsa-miR-126	
5	*MAPK11*	hsa-miR-122	
6	*MAPK14*	**hsa-miR-124**, hsa-miR-24	
7	*AKT1*	hsa-miR-125b, hsa-miR-149*, hsa-miR-185, hsa-miR-451	
8	*HRAS*	hsa-let-7a, hsa-miR-143, hsa-miR-181a	
9	*KRAS*	hsa-let-7a, hsa-let-7g, hsa-miR-143, hsa-miR-155, hsa-miR-181c, hsa-miR-18a*, hsa-miR-96	
10	*NRAS*	hsa-let-7a, hsa-let-7b, hsa-let-7c, hsa-miR-20a	
11	*RAF1*	hsa-miR-125b, **hsa-miR-7**	
12	*PPP3CA*	hsa-miR-145, hsa-miR-30a	
13	*PPP3R1*	hsa-miR-30a	
14	*NFAT5*	hsa-miR-24, hsa-miR-31	
15	*NFATC2*	hsa-miR-184	
16	*PTK2*	hsa-miR-193a-3p	
17	*RAC1*	hsa-miR-122, hsa-miR-194	
18	*CASP9*	hsa-let-7a, **hsa-miR-133a**	
19	*PTGS2*	hsa-let-7b, hsa-miR-101, hsa-miR-16, hsa-miR-26b	
20	*PLA2G4B*	**hsa-miR-338-3p**	
21	*MAPK1*	hsa-miR-199b-3p	
**PDGF Signaling Pathway (Biocarta)**			
**S. No.**	**Gene name**	**miRNA(s)**	
1	*PDGFA*	hsa-let-7d	
2	*PDGFRA*	hsa-let-7b,	
3	*GRB2*	**hsa-miR-433**	
4	*JAK1*	hsa-miR-17	
5	*RASA1*	hsa-miR-335	
6	*STAT1*	hsa-miR-145	
7	*STAT3*	hsa-miR-125b, hsa-miR-20b	
8	*STAT5A*	hsa-miR-222	
9	*SRF*	hsa-miR-122	
10	*FOS*	hsa-miR-101, **hsa-miR-221**, hsa-miR-222	
11	*JUN*	hsa-miR-30a	
12	*HRAS*	hsa-let-7a, hsa-miR-143, hsa-miR-181a,	
13	*RAF1*	hsa-miR-125b, **hsa-miR-7**	
14	*MAP2K1*	**hsa-miR-34a**, hsa-miR-424	
15	*MAP3K1*	hsa-miR-192	

## 9. miRNAs in Glioma Stem Cells (GSCs)

GSCs share properties with NPCs both in terms of cellular function and molecular mechanisms that govern these cellular functions. Expression levels of many key components of self-renewal machinery (e.g., Nestin, Bmi1, Olig2, Sox2 *etc*.) in NPCs are also expressed at high levels in GSCs [136]. NPCs during normal development are able to fully differentiate after exiting self-renewing state, GSCs on the other hand, appear to retain the self-renewal (indefinite proliferation) property while losing the capacity to terminally differentiate. MiRNAs like miR-124, miR-137, miR-128, miR-7 *etc*. are weakly expressed in gliomas and thus result in relieved repression of their targets (targets of these miRNAs include: CDK6, SCP1, PTBP1, LAMC1, ITGB1, BMI1, EGFR, E2F3A, IRS2 *etc*.). CDK6 a known promoter of G1/S phase transition has been shown to promote cell cycle in GSCs. Expression levels of has-miR-125b has been shown to be lower in GSCs and can target CDK6 [137]. BMI1 a polycomb group of genes, using mouse models it has been shown to maintain “stemness” of NSCs largely by blocking differentiation [138,139]. EGFR signaling has been shown to promote “stemness” of GSCs by activating expression of inhibitor of differentiation 3 (ID3) through SMAD5 [140]. The bone morphogenetic protein 4 (BMP4) regulated Smad signaling cascade responsible for inhibition of tumorigenic potential of GSCs [141] orchestrate their function in part by up-regulation of miR-451 [106]. This may explain sustained proliferation/self-renewal of GSCs [107,108,109,110]. CD133^+ve^ GSCs when compared to CD133^−ve^ glioma cells have lower expression levels of miR-425, miR-486 and miR-451 suggesting heterogeneous expression pattern for miRNAs within tumor. Activation of the Notch pathway has been implicated in the maintenance of GSCs (“stemness”, proliferation and radioresistance) [111,142,143,144]. Notch1 and Notch2 components of Notch pathway are targets of miR-34a, a miRNA which is down-regulated in GSCs [112]. Notch is also targeted by miR-326 in GSCs [113]. Thus miRNAs that play a crucial role in maintenance of self-renewal in normal NPCs have also been shown to promote undifferentiated status of GSCs.

## 10. miRNAs in Tumor Associated Angiogenesis

One of the most profound features of malignant progression of solid tumors is the induction of tumor neo-vascularization [145,146]. It is well established that regulation of miRNA expression is essential for normal development and differentiation of ECs and deregulated miRNAs can compromise vascular integrity in normal development. Multiple studies have revealed microRNA-driven regulation of angiogenesis in different tumors and this list is expanding rapidly. These miRNAs, which have been termed angiomiRs [147] may function by either promoting (pro-angiomiR) or inhibiting angiogenesis (anti-angiomiR), similar to pro- and anti-angiogenic factors that modulate different players of angiogenic pathways [147]. Some of the most studied pro-angiomiRs are miR-126, miR-17-92 cluster, miR-378, miR-296, miR-21, miR-210, miR-130a and some anti-angiomiRs such as miR-221/222 or miR-320 [114,115,116,117,118,119,120,121,122,123,124,127,128]. The role for miR-296 in glioma angiogenesis has been extensively studied in tumor and ECs [124]. MiR-296 was shown to be induced in glioma cells and endothelium through angiogenic growth factors and mediating angiogenesis *in vitro*. Furthermore, inhibition of miR-296 reduced neo-vascularization in subcutaneous tumors *in vivo* [124]. Recently another angiomiR, miR-125b was shown to down-regulate Myc-associated zinc finger protein (MAZ) and is also down regulated in GBMs. As MAZ is a known transcriptional activator of VEGF, reduction in MAZ indirectly activates VEGF and augments GBM angiogenesis [125]. Thus, modulating miR-125b may have therapeutic potential given its ability to inhibit vascularization. Similarly, miR-101 promotes glioma cell proliferation, migration, and angiogenesis *in vivo* and *in vitro* [126]. MiR-101 expression was reduced in GBM samples, resulting in increased expression of its target EZH2 and indirect induction of number of angiogenic genes [126]. Direct and indirect miRNA mediated mechanisms can regulate angiogenesis and tumor progression in GBMs.

## 11. miRNAs in Tumor Metabolism

Here we focus on microRNA-mediated regulation of aerobic glycolysis and/or signaling pathways that regulate glycolysis in gliomas and other cancers. Recently miR-451 was proposed as a regulator of the LKB1/AMPK signaling pathway in gliomas, which may be central to mechanisms that regulate cellular adaptation or resistance to metabolic stress during altered energy availability [129]. It has been shown that miR-326 can directly targets PKM2 isoform, which is highly overexpressed in cancer cells including GBMs, and can be utilized to regulate glioma metabolism [148]. The PI3K/AKT pathway regulates several aspects of glucose metabolism such as targeting and regulating glucose transporters or enhancing aerobic glycolysis through HK2 regulation. Several micro-RNAs have been shown to regulate PI3K/AKT pathway in gliomas such as oncogenic miR-21 and miR-26a, miR-221/222 as activators of AKT, and miR-451 and miR-128 as negative regulators of AKT pathway [149,150,151,152,153,154,155]. Tumor suppressor P53 has a role in dampening glycolysis and enhancing mitochondrial respiration, which is compatible with its tumor suppressor function. MiR-21 has been shown to regulate multiple important components of the P53 pathway in GBM cells. MiR-25 and miR-32 are part of a P53 mediated tumor suppressor circuitry [156,157]. When overexpressed in glioma cells they result in decreased cell growth and increased survival of xenograft models. Also, miR-34a was shown to have tumor suppressor function in P53-mutant U251 cells [158]. Recently, miR-155/143 cascade was shown to promote aerobic glycolysis indirectly by regulating HK2 expression [159]. This mechanism holds promise in GBMs since HK2 is highly expressed in GBMs as compared to normal brain or low-grade astrocytomas thus promoting aerobic glycolysis [160]. MYC regulation of glutamine and glucose metabolism is well established. MYC suppression by miR-23a/b and oncogenic function of miR-17-92 cluster has been shown in B cell lymphoma and prostate cancer to promote mitochondrial glutaminolysis, aerobic glycolysis, cell proliferation and might also be important to investigate in GBMs [130,131,161].

## 12. miRNA in Tumor Hypoxia

Low oxygen tension or cellular hypoxia has been shown to alter miRNA expression levels (up- or down-regulated). Conversely, miRNAs can regulate cellular HIF levels and its downstream genes resulting in plethora of effects including alterations in glucose transport, glucose metabolism, TCA cycle or angiogenesis. Amongst the HIF-dependent miRNAs known as hypoxamirs [162], miR210 has been extensively studied. MiR-210 is induced by HIF-1α [132] and was recently shown to be up-regulated in GBMs in hypoxic conditions *in-vitro* [163]. Also miR-210 was shown to repress mitochondrial metabolism under hypoxic conditions by decreasing expression of the iron-sulfur cluster assembly proteins ISCU1/2 [133]. This is in line with findings of Puisségur *et al*. regarding miR-210 mediated mitochondrial dysfunction by direct inhibition of succinate dehydrogenase subunit D (SDHD) and positive regulation of HIF by miR-210 (lung cancer). These functional effects mediated by miR-210 might have implications for altered metabolism in GBMs since it can modulate mitochondrial oxygen consumption and create a pseudohypoxic environment. Hypoxamirs such as miR-20b [134] in MCF-7 breast cancer cells and miR-199a in cardiomyocytes [135] can target HIF-1α to suppress its expression. Under hypoxic conditions miR-199a can directly target HIF-1α for inhibition, however under normoxia miR-199a derepresses and stabilizes HIF-1α by inhibiting sirtuin 1 (Sirt1), which in normal conditions can augments HIF signaling by inhibit prolyl hydroxylase 2 required for stabilization of HIF-1α [135]. Elaborate interactions of hypoxamirs and HIF-mediated responses add another level of complexity to hypoxic niche network. Deciphering hypoxamir regulation in different cellular context may shed a light on hypoxia-driven induction of aerobic glycolysis, angiogenesis, invasion and metastasis or stem cell phenotype in GBMs.

## 13. miRNA Mediated Networks in Glioma

Classically, miRNA and mRNA interaction is defined as a unidirectional regulation mechanism, where miRNA for the most part negatively regulates expression of target mRNA. This view of miRNA-mRNA interaction is challenged by competitive endogenous RNA hypothesis (ceRNA hypothesis). According to ceRNA hypothesis, mRNAs including RNAs arising from pseudogenes [164]) can negatively regulate miRNA function [165]. Over the recent years evidences for this mechanism in various cellular context (normal and cancer) has increased. Analysis of The Cancer Genome Atlas (TCGA) database revealed an extensive miRNA mediated RNA-RNA interaction network and explained mRNA function based on ceRNA hypothesis. PTEN tumor suppressor gene in glioma is discovered to be extensively integrated in the miRNA mediated RNA-RNA interaction network [166,167]. Through these newly identified miRNA-RNA networks, genes involved multiple pathways with known function in gliomagenesis are able to cross-talk and influence each other’s expression (RB, PTEN, PDGFR, VEGFA, RUNX1, and STAT3). Thus, miRNA apart from their widely reported function, are key participant in an extensive RNA-RNA interaction network and establish a new layer of epigenetic regulation. Since ceRNA mechanism rely on the relative abundance of RNAs (miRNAs and mRNAs) in the cells, specific interaction network may become dysfunctional, if concentration/expression of any of the interacting RNAs were to go to either extremes (*i.e*., no expression or over-expression). It is therefore conceivable that miRNAs in tumor have at least two distinct modes of action: (1) classical miRNA-mRNA regulation; (2) miRNAs in ceRNA-mediated interactions. This is very exciting and hopeful time in miRNA biology, however, the generation of miRNA mediated RNA-RNA interaction network based on physiologically relevant miRNA-mRNA interactions (rather than based bioinformatics prediction models) using biochemical assays and RNA sequencing is urgently needed. Thus, miRNA mediated mechanisms by connecting and establishing cross-talk between wide arrays of aberrant pathways and cellular functions may provide a global perspective to tackle heterogeneous and hard to manage tumors like GBM.

## 14. Conclusions

**Figure 1:**
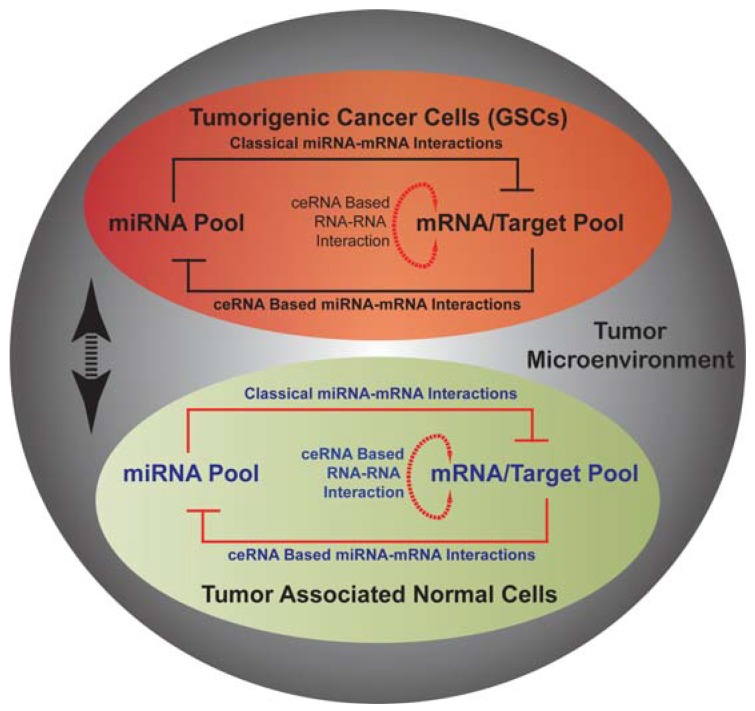
A schematic representation of possible miRNA mediated epigenetic network in tumorigenic cancer stem cells (GSCs; shown in black font color) and tumor associated normal cells (endothelial cells; shown in blue font color) within the tumor microenvironment. Double-headed black arrow represents cell-to-cell communication in tumor microenvironment and influence of extra-cellular factors on cellular functions.

Heterogeneous composition of GBMs with respect to resident tumor cells and parenchymal cells (EC, microglia, immune cells *etc*.) provides an additional layer of complexity to the pathology. The major limitations in the clinic are the multifactorial nature of the disease, its molecular and metabolic heterogeneity and the presence of functionally heterogeneous tumor cell populations. The differential expression of miRNA groups brought about by factors such as diverse cellular context, tumorigenic stages or the extent of metabolic stress pose a considerable challenge in therapy and evaluation of therapeutic effectiveness; making it less feasible to develop more personalized miRNA based treatment strategies at the present time. In this review we have discussed different interactions of tumor cells including GSCs and ECs with microenvironmental factors such as hypoxia and cellular metabolism, highlighting areas of interest for future investigations. At a cellular level, cancer cells (tumorigenic and non-tumorigenic) display a dynamic interaction within tumor (paracrine, autocrine, metabolic status, and cell-cell interactions) whereby creating a pro-tumor environment. As depicted in Figure 1, in cells within tumor including GSCs and ECs, an epigenetic program orchestrated by miRNA pool together with mRNA/RNA pool can explain multiple aspects of observed heterogeneity including metabolic and physiological heterogeneity. The interaction between various cell types at least in part is manifestation of their respective microRNA mediated epigenetic program. A newly described elaborate miRNA mediated RNA-RNA network can explain multiple sources of molecular diversity, which ultimately results in a tumor with heterogeneous features. A better understanding of this epigenetic program may provide novel methods for management of GBM patients.
